# High Level Expression of MHC-II in HPV+ Head and Neck Cancers Suggests that Tumor Epithelial Cells Serve an Important Role as Accessory Antigen Presenting Cells

**DOI:** 10.3390/cancers11081129

**Published:** 2019-08-07

**Authors:** Steven F. Gameiro, Farhad Ghasemi, John W. Barrett, Anthony C. Nichols, Joe S. Mymryk

**Affiliations:** 1 Department of Microbiology and Immunology, The University of Western Ontario, London, ON N6A 3K7, Canada; 2 Department of Otolaryngology, Head & Neck Surgery, The University of Western Ontario, London, ON N6A 3K7, Canada; 3 Department of Oncology, The University of Western Ontario, London, ON N6A 3K7, Canada; 4 London Regional Cancer Program, Lawson Health Research Institute, London, ON N6C 2R5, Canada

**Keywords:** human papillomavirus, MHC-II, major histocompatibility complex, antigen presentation, head and neck carcinoma, co-stimulatory molecules, survival signals, T-cell

## Abstract

High-risk human papillomaviruses (HPVs) are responsible for a subset of head and neck squamous cell carcinomas (HNSCC). Expression of class II major histocompatibility complex (MHC-II) is associated with antigen presenting cells (APCs). During inflammation, epithelial cells can be induced to express MHC-II and function as accessory APCs. Utilizing RNA-seq data from over 500 HNSCC patients from The Cancer Genome Atlas, we determined the impact of HPV-status on the expression of MHC-II genes and related genes involved in their regulation, antigen presentation, and T-cell co-stimulation. Expression of virtually all MHC-II genes was significantly upregulated in HPV+ carcinomas compared to HPV− or normal control tissue. Similarly, genes that encode products involved in antigen presentation were also significantly upregulated in the HPV+ cohort. In addition, the expression of *CIITA* and *RFX5*—regulators of MHC-II—were significantly upregulated in HPV+ tumors. This coordinated upregulation of MHC-II genes was correlated with higher intratumoral levels of interferon-gamma in HPV+ carcinomas. Furthermore, genes that encode various co-stimulatory molecules involved in T-cell activation and survival were also significantly upregulated in HPV+ tumors. Collectively, these results suggest a previously unappreciated role for epithelial cells in antigen presentation that functionally contributes to the highly immunogenic tumor microenvironment observed in HPV+ HNSCC.

## 1. Introduction

High-risk human papillomaviruses (HPVs) are small, non-enveloped, double-stranded DNA viruses that are responsible for an estimated 5% of all human cancers [1,2]. These biological carcinogens are the causative agents of virtually all cervical cancers and a subset of head and neck squamous cell carcinomas (HNSCC) [1]. HNSCC are a heterogenous group of malignancies caused by multiple distinct etiologies. Infection with high-risk HPVs is responsible for approximately 85,000 of the 600,000 global annual cases of HNSCC, making it the second most common cause of HPV-induced cancers [3,4,5]. HPV-positive (HPV+) tumors are distinct from their HPV-negative (HPV−) counterparts from a molecular perspective, with distinct genetic, epigenetic, and protein expression profiles [6,7,8,9,10,11]. Interestingly, patients with HPV+ HNSCC tumors have markedly better clinical outcomes compared to those with HPV− tumors, leading to the recognition of HPV+ HNSCC as unique clinical entities [12,13,14]. 

We and others have noted significant differences in the immune landscape between the tumor microenvironments of HPV+ and HPV− HNSCC [7,9,15,16,17,18,19]. Specifically, HPV+ tumors express higher levels of class I major histocompatibility complex (MHC-I) compared to their HPV− counterparts, which could be a consequence of the higher intratumoral levels of interferon-gamma (IFNγ) observed in the tumors of the HPV+ cohort [9]. Utilizing an immunogenomic approach, we have also shown that HPV+ HNSCC tumors exhibited a strong Th1 response characterized by increased infiltration with multiple types of T-cells—CD4+, CD8+, and regulatory T-cells—and expression of their effector molecules [7]. In addition, HPV+ HNSCC also expressed higher levels of *CD39* and multiple T-cell exhaustion markers including *LAG3*, *PD1*, *TIGIT*, and *TIM3* compared to HPV− HNSCC. Importantly, patients with higher expression of these exhaustion markers—indicative of a T-cell-inflamed tumor—exhibited markedly improved survival in HPV+, but not HPV−, HNSCC [7].

In order for an effective T-cell-specific anti-tumor response to occur, a tumor associated antigen must be presented in either the context of MHC-I or class II MHC (MHC-II) [20]. This process is dependent on the initial acquisition of specific antigenic peptides by surveilling antigen presenting cells (APCs). APCs present these exogenous peptides on their cell surface in the context of major histocompatibility complex-II (MHC-II) to activate cognate CD4+ helper T-cells in an antigen-specific fashion [21]. This crosslinking of T-cell receptor (TCR) with its cognate antigen-MHC-II complex is the initial step in the activation of T-cells. The next step is the crosslinking of co-stimulatory molecules between T-cell and APCs that will provide the appropriate signals to initiate T-cell proliferation and survival [22]. Once activated, CD4+ T-cells then stimulate the proliferation of CD8+ cytotoxic T-cells (CTLs) that recognize and respond to the initial antigenic peptide. CTLs subsequently target tumor cells for lysis based on presentation of the cognate endogenously derived antigenic peptides on the tumor cell surface in the context of MHC-I [23,24].

Although the ability of HPV to suppress MHC-I expression in cell culture systems is well known [25,26], this is not likely the case in actual human tumors. Indeed, we previously reported that HPV+ head and neck cancers express higher levels of MHC-I than HPV− tumors [9]. Thus, HPV+ tumor cells may be more effective at displaying endogenously derived viral or neo-antigenic peptides, making them more easily targeted for CTL lysis. Expression of MHC-II molecules is typically restricted to professional APCs, such as dendritic cells (DCs), macrophages, and B-cells [27]. However, epithelial cells can be stimulated by proinflammatory cytokines—specifically IFNγ—to express MHC-II and function as accessory APCs to stimulate T-cell responses [28,29,30]. As mentioned above, MHC-II proteins play a key role in presenting exogenously-derived peptide antigens that ultimately lead to an effective CTL response, and it is likely that the induced tumor-specific MHC-II expression on epithelial cells may accentuate this process. Indeed, the role of tumor cell derived MHC-II in anti-tumor immunity has become increasingly appreciated [31], with accumulating evidence suggesting that tumor-specific MHC-II expression is correlated with favorable outcomes in melanoma, classic Hodgkin’s lymphoma, breast cancer, and oropharyngeal cancers [31,32,33,34,35].

In this study, we used RNA-seq data from over 500 human head and neck tumors from The Cancer Genome Atlas (TCGA) to determine if the presence of oncogenic HPV is associated with altered expression of all classical MHC-II genes and other key genes involved in their regulation, antigen presentation, and T-cell co-stimulation. We found that expression of virtually all classical MHC-II genes was significantly upregulated in HPV+ tumors compared to their HPV− counterparts or normal control tissue. Similarly, genes that encode products that are fundamental to proper antigen loading and presentation were also significantly upregulated in HPV+ tumors. Importantly, the relative level of expression of these inducible MHC-II genes was far beyond those genes associated with professional APCs. Furthermore, the expression of *class II major histocompatibility complex transactivator* (*CIITA*) and *regulatory factor X5* (*RFX5*)—essential master regulators of the MHC-II transcriptional control system—were significantly upregulated in the HPV+ cohort. This coordinated upregulation of the mRNA levels of genes involved in the MHC-II antigen presentation pathway and their regulation were correlated with the higher intratumoral levels of IFNγ observed in HPV+ carcinomas. In addition, genes that encode various T-cell co-stimulatory molecules involved in T-cell activation and survival were found to be significantly upregulated in HPV+ tumors compared to HPV− tumors and normal control tissue. Taken together, these results suggest a previously unappreciated role for epithelial cells in antigen presentation that functionally contributes to the highly immunogenic tumor microenvironment observed in HPV+ HNSCC. This further illustrates the profound differences in the immune landscape between the tumor microenvironments of HPV+ and HPV− HNSCC and may in part contribute to the superior clinical outcomes that are associated with HPV+ HNSCC.

## 2. Results

### 2.1. Classical MHC Class II α- and β-Chain Genes are Expressed at Higher Levels in HPV+ Head and Neck Carcinomas 

**Figure 1:**
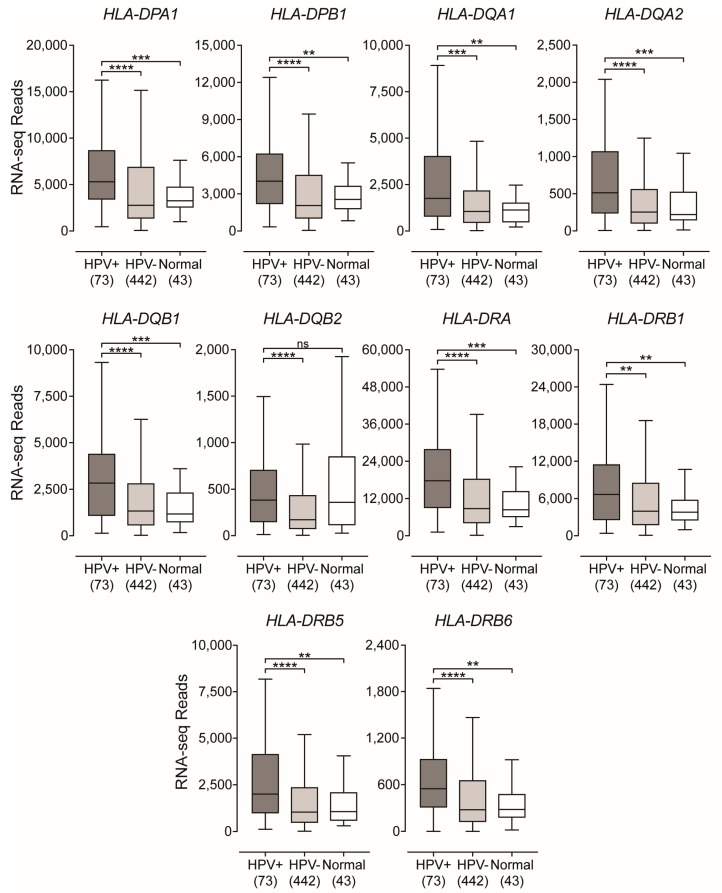
Expression of classical class II major histocompatibility complex (MHC-II) α- and β-chain genes in head and neck squamous cell carcinomas stratified by high-risk human papillomaviruses (HPV)-status. RNA-Seq by Expectation Maximization (RSEM)-normalized RNA-seq data for the indicated MHC-II genes was extracted from The Cancer Genome Atlas (TCGA) database for the head and neck squamous cell carcinoma (HNSC) cohort for HPV+, HPV−, and normal control tissues. Numbers in brackets refer to the number of samples included in each analysis. Statistical analysis was performed using a two-tailed non-parametric Mann–Whitney U test. ** *p* ≤ 0.01, *** *p* ≤ 0.001, **** *p* ≤ 0.0001, ns—not significant.

Constitutive expression of classical MHC-II molecules is typically restricted to professional antigen presenting cells—DCs, macrophages, and B-cells [27]. However, in non-immune cells that lack constitutive expression, such as those of the epithelia, their expression can be induced through exposure to proinflammatory cytokines [28,29,36]. In humans, the genes that encode the three classical polymorphic MHC-II molecules HLA-DP, HLA-DQ, and HLA-DR are expressed as α- and β-chains that form heterodimers on the cell surface [25]. We began by analyzing the Illumina HiSeq RNA expression data from the TCGA HNSC cohort for expression of *HLA-DPA1*, *-DPB1*, *-DQA1*, *-DQA2*, *-DQB1*, *-DQB2*, *-DRA*, *-DRB1*, *-DRB5*, and *-DRB6* genes, encoding the various α- and β-chains for all three classical isotypes (Figure 1). Uniformly, all HPV+ patient samples expressed significantly higher levels of mRNA for all 10 MHC-II genes analyzed compared to HPV− patient samples and virtually all normal control tissues—with the exception of *HLA-DQB2* (HPV+ versus Normal). As the majority of the HPV+ samples are from the oropharynx subsite, we repeated this analysis with HPV+ and HPV− samples that occur only in the oropharynx. Similarly, to the analysis including all subsites, HPV+ oropharyngeal tumors expressed significantly higher levels of MHC-II α- and β-chain genes compared to their HPV− oropharyngeal tumor counterparts (Supplementary Materials Figure S1). Collectively, these results indicate that HPV+ head and neck tumors express high levels of the mRNAs encoding the α- and β-chain heterodimers of the classical MHC-II molecules versus HPV− tumors or normal control tissues. It is noteworthy that based on the normalized read levels, all of these genes are expressed at levels several orders of magnitude above any markers of professional APCs, such as *CD19* (B-cells) [37], *CCL13* (DCs) [38], and *CD84* (macrophages) [39] (Supplementary Materials Figure S2A). However, these normalized read levels are comparable to that of an established epithelial cell marker, E-cadherin (*CDH1*) [40] (Supplementary Materials Figure S2B). Thus, it is likely that these genes are being expressed by epithelial cells within the actual tumor.

### 2.2. Genes Encoding Key Components of the MHC-II Antigen Presentation Pathway are Expressed at Higher Levels in HPV+ Head and Neck Carcinomas 

**Figure 2:**
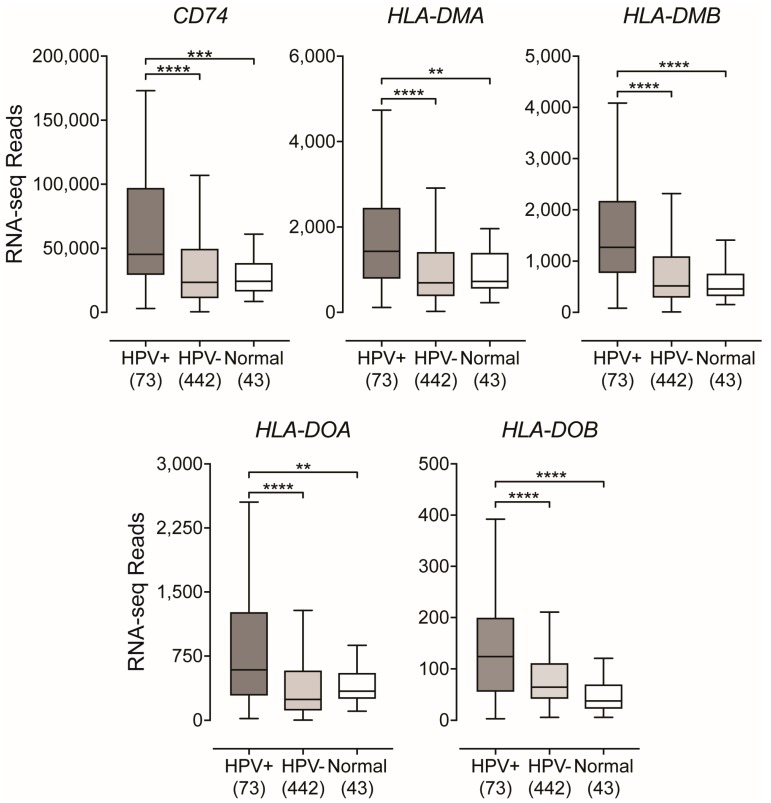
Expression of the invariant chain and MHC class II-like genes in head and neck carcinomas stratified by HPV-status. RSEM normalized RNA-seq data for the indicated genes involved in MHC-II-dependent antigen processing and presentation was extracted from the TCGA database for the HNSC cohort for HPV+, HPV−, and normal control tissues. Numbers in brackets refer to the number of samples included in each analysis. Statistical analysis was performed using a two-tailed non-parametric Mann–Whitney U test. ** *p* ≤ 0.01, *** *p* ≤ 0.001, **** *p* ≤ 0.0001, ns—not significant.

In the endoplasmic reticulum (ER), newly synthesized MHC-II α- and β-chains form a trimeric complex with a non-polymorphic protein called the invariant chain (Ii), which is encoded by the *HLA-DR antigens-associated invariant chain* or *Cluster of Differentiation 74* (*CD74*) gene [41]. This association with the Ii chain prevents premature peptide loading and also dictates the trafficking of the Ii-MHC-II complex to the endosomal-lysosomal antigen-processing compartments, which contain the antigenic peptides [21,42]. Once in this compartment, Ii is proteolytically cleaved, leaving only a small fragment in the peptide-binding groove called the class II-associated invariant chain peptide (CLIP). Similarly, to the genes encoding the classical MHC-II α- and β-chains, *CD74* was found to be significantly upregulated in HPV+ HNSCC compared to their HPV− counterparts or normal control tissues (Figure 2).

In order for antigenic peptide-binding to occur, CLIP must be removed from the peptide-binding groove [21,41]. The enzymatic removal of CLIP is mediated by the MHC class II-like heterodimer, HLA-DM. After HLA-DM-mediated removal of CLIP, the class II molecules can now bind lysosomally generated antigenic peptides [43,44]. The binding of antigenic peptides is influenced by another MHC class II-like heterodimer, HLA-DO, which regulates the MHC-II peptide repertoire by modulating the activity of HLA-DM [45,46]. The α- and β-chains of these dimeric class II-like molecules are encoded by the *HLA-DMA*, *HLA-DMB*, *HLA-DOA*, and *HLA-DOB* genes. Like the classical MHC-II genes, all four genes encoding the class II-like MHC molecules are similarly upregulated in HPV+ HNSCC versus HPV− tumors or normal control tissues (Figure 2).

When repeated only considering the oropharynx subsite, HPV+ oropharyngeal tumors expressed significantly higher levels of the invariant chain and MHC-II-like genes compared to their HPV− oropharyngeal tumor counterparts (Supplementary Materials Figure S1). Taken together, the upregulation of the genes encoding the MHC-II invariant chain and class II like genes, suggests that key components of the MHC-II antigen presentation pathway are transcribed in HPV+ HNSCC at levels that are significantly higher than observed in HPV− tumors or normal control tissues. Furthermore, these genes are also expressed at very high levels, with the exception of *HLA-DOB*, that are indicative of being expressed by epithelial cells within the actual tumor.

### 2.3. Impact of HPV-Status on the Expression of Transcriptional Regulators of MHC-II Gene Expression

**Figure 3:**
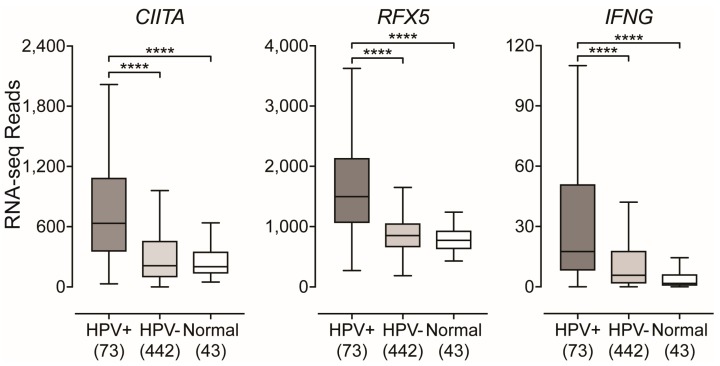
Expression of *class II major histocompatibility complex transactivator* (*CIITA)*, *regulatory factor X5* (*RFX5)*, *and interferon-gamma* (*IFNG)* mRNA in head and neck carcinomas stratified by HPV-status. RSEM normalized RNA-seq data for the *CIITA*, *RFX5*, and *IFNG* genes were extracted from the TCGA database for the HNSC cohort for HPV+, HPV−, and normal control tissues. Numbers in brackets refer to the number of samples included in each analysis. Statistical analysis was performed using a two-tailed non-parametric Mann–Whitney U test. ** *p* ≤ 0.01, *** *p* ≤ 0.001, **** *p* ≤ 0.0001, ns—not significant.

Transcriptional control of MHC-II genes and the related genes that encode key components of the MHC-II antigen presentation pathway is among one of the best understood systems in mammals. It is a complex transcriptional system with a unique method of regulation that is completely dependent on the master transcriptional regulator CIITA [29,47]. In agreement with the high levels of MHC-II genes and related genes, analysis of the TCGA data reveals significantly higher levels of *CIITA* in HPV+ samples compared to HPV− or normal control tissues (Figure 3). In addition, *RFX5*—another important transcriptional regulator of MHC-II genes [29]—was similarly expressed at significantly higher levels in HPV+ samples with respect to HPV− tumors or normal control tissues (Figure 3). Again, these differences were also observed when only considering the expression of these genes in the oropharynx (Supplementary Materials Figure S1).

Expression of MHC-II genes and related genes are restricted to APCs, however non-hematopoietic cells such as fibroblasts, endothelial cells, and epithelial cells can be stimulated by IFNγ to express MHC-II molecules and related proteins involved in the antigen presentation pathway [28,29,36]. We analyzed the expression level of the IFNγ gene (*IFNG*) (Figure 3). As expected, the relative level of *IFNG* expression was similar in magnitude to that of other leukocyte specific genes (Supplementary Materials Figure S2A). However, *IFNG* was expressed at significantly higher levels in HPV+ tumors compared to its HPV− counterpart or normal control tissues (Figure 3). In addition, when only considering the oropharynx, the expression of the IFNγ gene was significantly higher in HPV+ samples compared to HPV− oropharyngeal tumors (Supplementary Materials Figure S1).

**Figure 4:**
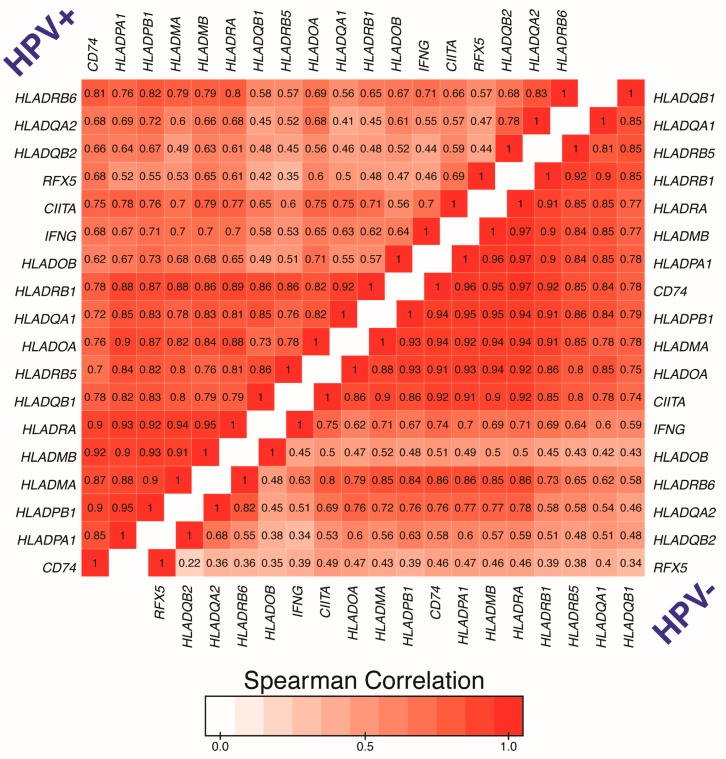
Correlation matrix of genes involved in the MHC-II antigen presentation pathway in head and neck carcinomas stratified by HPV-status. RSEM normalized RNA-seq data for the genes listed above were extracted from the TCGA database for the HNSC cohort for HPV+ (upper triangle) and HPV− samples (lower triangle). Pairwise spearman correlation was performed followed by hierarchical clustering to group based on correlation. Number in boxes indicate Spearman’s rank correlation coefficient of analyzed gene pairs.

To further illustrate the IFNγ-specific coordinated upregulation of MHC-II genes and related genes that encode products essential for antigen processing and presentation, we generated a correlation matrix for both HPV+ (Figure 4: upper triangle) and HPV− samples (Figure 4: lower triangle). As expected, regardless of HPV-status, we found that expression of all MHC-II antigen presentation-specific genes were statistically correlated in a pairwise fashion in each patient sample (see also Supplementary MaterialsTable S1).

Thus, the upregulated expression of *CIITA*, *RFX5*, and subsequent expression of all the classical MHC-II genes and related genes required for antigen loading and presentation observed in HPV+ head and neck carcinomas are likely a consequence of IFNγ exposure. Furthermore, the correlation matrix illustrates the unique simultaneous coordination of the MHC-II transcriptional control system that has been shown to be dictated by the master transcriptional regulator CIITA [29,47]. 

### 2.4. Impact of HPV-Status on the Expression of T-Cell Co-Stimulatory Molecules in HPV-Positive Head and Neck Carcinomas

**Figure 5:**
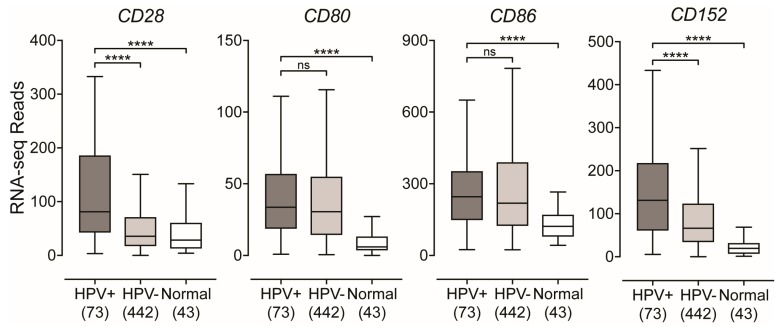
Expression of genes that encode for T-cell co-stimulatory molecules in head and neck carcinomas stratified by HPV-status. RSEM normalized RNA-seq data for genes that encode T-cell specific co-stimulatory molecules was extracted from the TCGA database for the HNSC cohort for HPV+, HPV−, and normal control tissues. Statistical analysis was performed using a two-tailed non-parametric Mann–Whitney U test. Numbers in brackets refer to the number of samples included in each analysis. **** *p* ≤ 0.0001, ns—not significant.

Co-stimulation of T-cells occurs through the interaction of its constitutively expressed CD28 receptor with either CD80 or CD86 on APCs [22]. Utilizing RNA expression data for the levels of each of these co-stimulatory molecules, we found higher levels of *CD28* in HPV+ tumors compared to HPV− or normal control tissues (Figure 5). While the levels of both *CD80* and *CD86* in HPV+ tumors were not significantly different compared to their HPV− counterparts, they were significantly higher compared to normal control tissues (Figure 5). In addition, we found that the mRNA levels of *CD152*, which encodes for CTLA-4, a marker of T-cell activation [48], was significantly upregulated in HPV+ tumors compared to HPV− and normal control tissues (Figure 5). Again, these differences were also observed when only considering the expression of these genes in the oropharynx (Supplementary Materials Figure S1). These results suggest that, like the MHC-II genes and the genes involved in antigen loading and presentation, co-stimulatory molecules are similarly present at higher levels in HPV+ tumors and this is correlated with a higher level of T-cell activation.

### 2.5. Impact of HPV-Status on the Expression of Inducible T-cell Survival Signal Molecules in HPV-Positive Head and Neck Carcinomas

**Figure 6:**
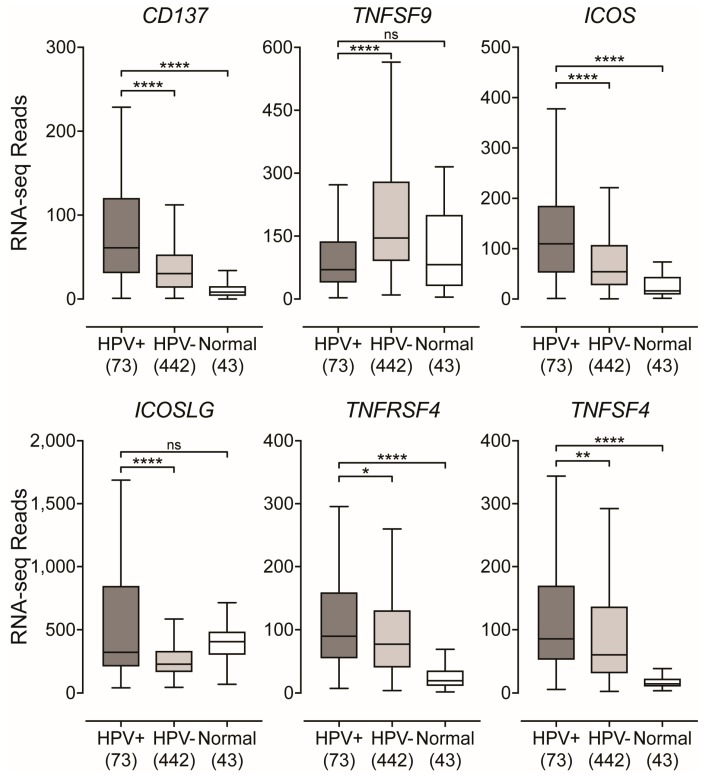
Expression of inducible T-cell survival signal molecules in head and neck carcinomas stratified by HPV-status. RSEM normalized RNA-seq data for inducible genes that encode for T-cell survival molecules was extracted from the TCGA database for the HNSC cohort for HPV+, HPV−, and normal control tissues. Statistical analysis was performed using a two-tailed non-parametric Mann–Whitney U test. Numbers in brackets refer to the number of samples included in each analysis. * *p* ≤ 0.05, ** *p* ≤ 0.01, *** *p* ≤ 0.001, **** *p* ≤ 0.0001, ns—not significant.

Utilizing the RNA-seq HNSC dataset from the TCGA, we looked at genes that encode for inducible, T-cell activation-dependent, survival signal molecules and their respective ligands [22,49]. We found that *CD137* (4-1BB, TNFRSF9) was significantly upregulated in HPV+ tumors compared to HPV− or normal control tissues (Figure 6). However, its ligand *TNFSF9* (CD137L, 4-1BBL) was found to be significantly downregulated in HPV+ tumors compared to HPV− and not significantly different compared to normal control tissues (Figure 6). Next, we looked at the genes that encode for the inducible T-cell co-stimulator (*ICOS*) and its ligand *ICOSLG* and found that both were significantly upregulated in HPV+ tumors compared to HPV−, but only *ICOS* was significantly upregulated in comparison to normal control tissues (Figure 6). Finally, we looked at OX40 (*TNFRSF4*, CD134) and its ligand OX40L (*TNFSF4*, CD252) and found that both genes were significantly upregulated in HPV+ tumors compared to their HPV− counterparts and normal control tissues (Figure 6). Again, these differences were also observed when only considering the expression of these genes in the oropharynx with the exception of *TNFSF4* (Supplementary Materials Figure S1). Taken together, these results suggest that T-cells are activated and proliferating within the HPV+ tumor microenvironment via the observation of an increase in expression of genes that encode for survival signal molecules that are only induced following TCR-mediated antigen-specific T-cell activation and/or CD28 co-stimulation [22,49].

## 3. Discussion

Expression of MHC-II is typically associated with professional APCs, which are considered essential for the initiation of the adaptive immune response. They function by sampling their local environment via phagocytosis, acquiring particles and processing them internally in order to present them on their cell surface to CD4+ T-cells in the context of an antigen-MHC-II complex [21]. The crosslinking of the CD4+ TCR and antigen-MHC-II complex initiates the T-cell activation protocol that, in conjunction with co-stimulatory signals, can ultimately lead to an effective adaptive immune response against a threat of internal or external origin, such as cancerous cells or bacteria and viruses, respectively [22].

In non-hematopoietic cells, such as epithelia, MHC-II expression can be induced through exposure to the pro-inflammatory cytokine IFNγ [28,29,36]. This induction of MHC-II on epithelial cells bestows on them the ability to act as accessory APCs, and this can accentuate the presentation of antigens to CD4+ T-cells [30]. However, the ability of epithelial cells to function as accessory APCs is generally underappreciated, with most existing information related to the gastrointestinal and respiratory tracts [50]. Interestingly, cancerous tissues can retain tumor-specific MHC-II expression, and this has the potential to increase recognition of a tumor by the immune system [31]. Indeed, tumor specific MHC-II expression has been associated with superior prognosis and/or improved response to immune checkpoint inhibitor therapy in several human cancers, as well as increased tumor rejection in murine models [31,32,33,34,35]. The importance of the immune response for successful resolution of cancers cannot be understated. Indeed, mouse models have shown that neither chemotherapy nor radiation treatment functioned effectively in the absence of a functional immune system, at least in HPV+ HNSCC [51].

While MHC-II expression has been reported in head and neck tumors [35], existing studies have been limited to individual classical isotypes, such as HLA-DR, based on limitations in available antibodies [52]. Cell culture models based on established head and neck cancer lines have also demonstrated MHC-II expression, often in response to IFNγ, or transfection with CIITA—master regulator of MHC-II transcription [53,54]. However, no existing studies have comprehensively assessed the transcriptional status of the entire MHC-II antigen presentation system in head and neck cancers. In this study, our goal was to determine if MHC-II components were widely expressed in human head and neck cancers and whether expression was influenced by HPV-status.

Using data from over 500 primary human head and neck tumors, we provide evidence that HPV+ head and neck carcinomas display high mRNA levels for virtually all MHC-II genes, including the classical and non-classical α- and β-chains, the invariant γ chain, as well as factors required for MHC-II loading and trafficking (Figure 1 and Figure 2). Increased expression of these genes was observed whether the comparison included all head and neck cancer subsites or was restricted to just the oropharynx, where the majority of HPV+ head and neck cancers arise (Supplementary Materials Figure S1). These results are in good agreement with the concurrent detection of high levels of expression of *CIITA* and *RFX5*, important global regulators of MHC-II transcription [29,47], in HPV+ tumors. HPV+ head and neck carcinomas express significantly higher levels of these genes as compared to normal control tissues, and these levels are generally higher than in HPV− carcinomas (Figure 3). This likely reflects the T-cell inflamed nature of HPV+ cancers [7], and specifically the higher levels of IFNγ expressed in these tumors (Figure 3). This IFNγ-dependent coordinated upregulation of MHC-II genes and related genes involved in antigen processing and presentation was further illustrated in Figure 4, where we observed a strong global correlation with all genes analyzed in the MHC-II transcriptional control system.

After generation and programming in the thymus, CD8+ and CD4+ T-cells circulate in the body until they encounter their specific antigen presented on either class I or class II MHC molecules, respectively [23,24,55]. This interaction between TCR and antigen-loaded MHC complex represents signal 1, which triggers the activation of T-cells. However, in order for the activated T-cell to fully respond to the presented threat, and not enter a state of unresponsiveness, it requires a secondary signal through co-stimulatory molecules [22]. We found higher levels of *CD28* in HPV+ tumors compared to their HPV− counterparts and normal control tissues (Figure 5). The binding of CD28 with either CD80 or CD86 leads to clonal expansion of the T-cell pool that is specific to the recognized antigen [22,49]. In order to attenuate this response, the aforementioned interaction leads to the induction of the co-inhibitory molecule CTLA-4, which is encoded by the gene *CD152*. This co-inhibitory molecule will compete with CD28 for binding to either CD80 or CD86 to attenuate the T-cell response [22,48]. We found that *CD152* was significantly upregulated in the HPV+ cohort (Figure 5). Collectively, this data indirectly illustrates the higher number of infiltrating T-cells within the tumor microenvironments of HPV+ HNSCC through the identification of significantly higher levels of the constitutively expressed T-cell-specific *CD28* marker. In addition, it provides good evidence that the interaction of co-stimulatory molecules in HPV+ HNSCC is effective, given that we detected significantly higher levels of expression of *CD152* mRNA, which encodes for the inducible co-inhibitory molecule CTLA-4.

In order for proliferating T-cells to persist and survive after antigen-recognition and subsequent stimulation with co-stimulatory molecules, they require survival signals that are delivered through the cross-linking of various molecules [22,49]. Interestingly, unlike other co-stimulatory molecules—such as CD28—that can be found constitutively expressed on T-cells, the molecules that convey these survival signals are encoded by genes that are only expressed following TCR-mediated antigen-specific T-cell activation and/or CD28 co-stimulation [22,49]. We found that all inducible T-cell survival genes analyzed, with the exception of *TNFSF9*, were expressed at significantly higher levels in HPV+ tumors compared to HPV− and normal control tissues (Figure 6). *TNFSF9* was significantly downregulated in HPV+ tumors compared to its HPV− counterpart (Figure 6). This downregulation of *TNFSF9* is indicative of a mechanistic response to excessive CD137-mediated signaling [56,57,58]. This data indirectly illustrates that the HPV+ tumor microenvironment contains increased levels of activated and proliferating T-cells via the observation of an increase in expression of genes that encode for survival signal molecules that are induced following TCR-mediated antigen-specific T-cell activation and/or CD28 co-stimulation. These results agree well with previous reports by our group and others that HPV+ tumors contain more T-cells [7,16,17,18,19], but goes further in that it indirectly provides evidence of productive MHC-II-dependent tumor-antigen recognition.

## 4. Materials and Methods 

### 4.1. TCGA RNA-Seq Boxplot Comparisons

Level 3 RNA-Seq by Expectation Maximization (RSEM)-normalized Illumina HiSeq RNA expression data for the TCGA head and neck cancer (HNSC) cohort was downloaded from the Broad Genome Data Analysis Centers Firehose server (https://gdac.broadinstitute.org/). The normalized, gene level Firehose dataset was utilized for all the genes analyzed. RSEM-normalized RNA-Seq data was extracted into Microsoft Excel and the HPV-status was determined based on published datasets [59,60,61,62]. For all genes analyzed in this study, patient samples from primary tumors with known HPV-status were grouped as HPV+, HPV−, or normal control samples. Patient samples with undetermined HPV-status or samples from secondary metastatic lesions were omitted from our calculations. This resulted in 73 HPV+, 442 HPV−, and 43 normal control samples with RNA-Seq data available for gene expression analysis. Oropharynx-only gene reanalysis was performed by utilizing patient samples that were isolated from the tissues of the oropharynx (tonsils, base of tongue, or oropharynx) for both HPV+ and HPV− samples. This resulted in 53 HPV+ and 26 HPV− samples with RNA-Seq data available for gene expression analysis. Graphpad Prism v7.0 (Graphpad Software, Inc., San Diego, California, USA) was used to generate boxplot comparisons of gene expression between the indicated HPV+, HPV−, and normal control samples as well as the oropharynx-only reanalysis between HPV+ and HPV− samples. For each boxplot, the center line indicates the median, the lower and upper box limits represent Q1 (25th percentile) and Q3 (75th percentile), respectively, and the whiskers extend 1.5 times the interquartile range (IQR) from Q1 (lower whisker) and Q3 (upper whisker). An unpaired, two-tailed non-parametric Mann–Whitney U test was utilized to assign p-values. G*Power Software version 3.1.9.2 [63] was used to perform post-hoc power calculations, with effect size selected as 0.8 and α = 0.05. All boxplot comparisons achieved a power value > 0.8. Figures were assembled into final form using Adobe Illustrator CS6.

### 4.2. Correlation Matrix

Level 3 RSEM normalized RNA-seq data for the genes listed above were extracted from the TCGA database and processed as detailed in Materials and Methods 4.1. For the HPV+ (upper triangle) and HPV− samples (lower triangle), pairwise spearman correlation was performed for each gene involved in the MHC-II transcriptional control system. Hierarchical clustering was utilized to group genes based on strength of correlation. Correlations and clustering were performed using R statistical environment (version 3.4.0; https://cran.r-project.org/bin/macosx/) utilizing packages ggplot2 and reshape2. Correlation matrix figure was assembled into final form using Adobe Illustrator.

## 5. Conclusions

HPV+ HNSCC tumors are remarkably different from their HPV− counterparts in that they express high levels of all components of the MHC-II antigen presentation apparatus. Importantly, while some of this signal can be attributed to professional APCs, the extremely high relative level of expression supports a model whereby the T-cell inflamed environment in HPV+ HNSCC induces a functionally effective MHC-II presentation system based on tumor epithelial cells. As MHC-II-dependent antigen presentation is critical for CD4+ help in CD8+ T-cell responses, which are essential for the control and clearance of cancerous cells, it is likely that the expression of non-self-derived viral antigens or tumor derived neoantigens, combined with intact MHC-II presentation and appropriate co-stimulation contributes to the markedly better patient outcomes for HPV+ versus HPV− head and neck carcinomas. As immune checkpoint inhibition therapy in other cancers has been reported to be most effective for tumors with high MHC-II levels, suggesting that stratification based on MHC-II levels may help predict those likely to respond to checkpoint inhibition therapy.

## Supplementary Materials

The following are available online at https://www.mdpi.com/2072-6694/11/8/1129/s1, Figure S1: Gene expression reanalysis in the oropharynx. Figure S2: Gene expression comparisons of (A) APC and (B) Epithelia markers. Table S1: MHC-II genes pairwise correlation, Spearman coefficients, and P-values.
