# Supplementary material for: High Level Expression of MHC-II in HPV+ Head and Neck Cancers Suggests that Tumor Epithelial Cells Serve an Important Role as Accessory Antigen Presenting Cells
Source: Cancers (Basel). 2019 Aug 7;11(8):1129. doi: 10.3390/cancers11081129 (PMC6721589; doi:10.3390/cancers11081129)
Supplement: Supplementary file 1 [file cancers-11-01129-s001.zip › Supplementary Materials Figure S1, Figure S2, and Table S1/Supplementary Figure 2.pdf]

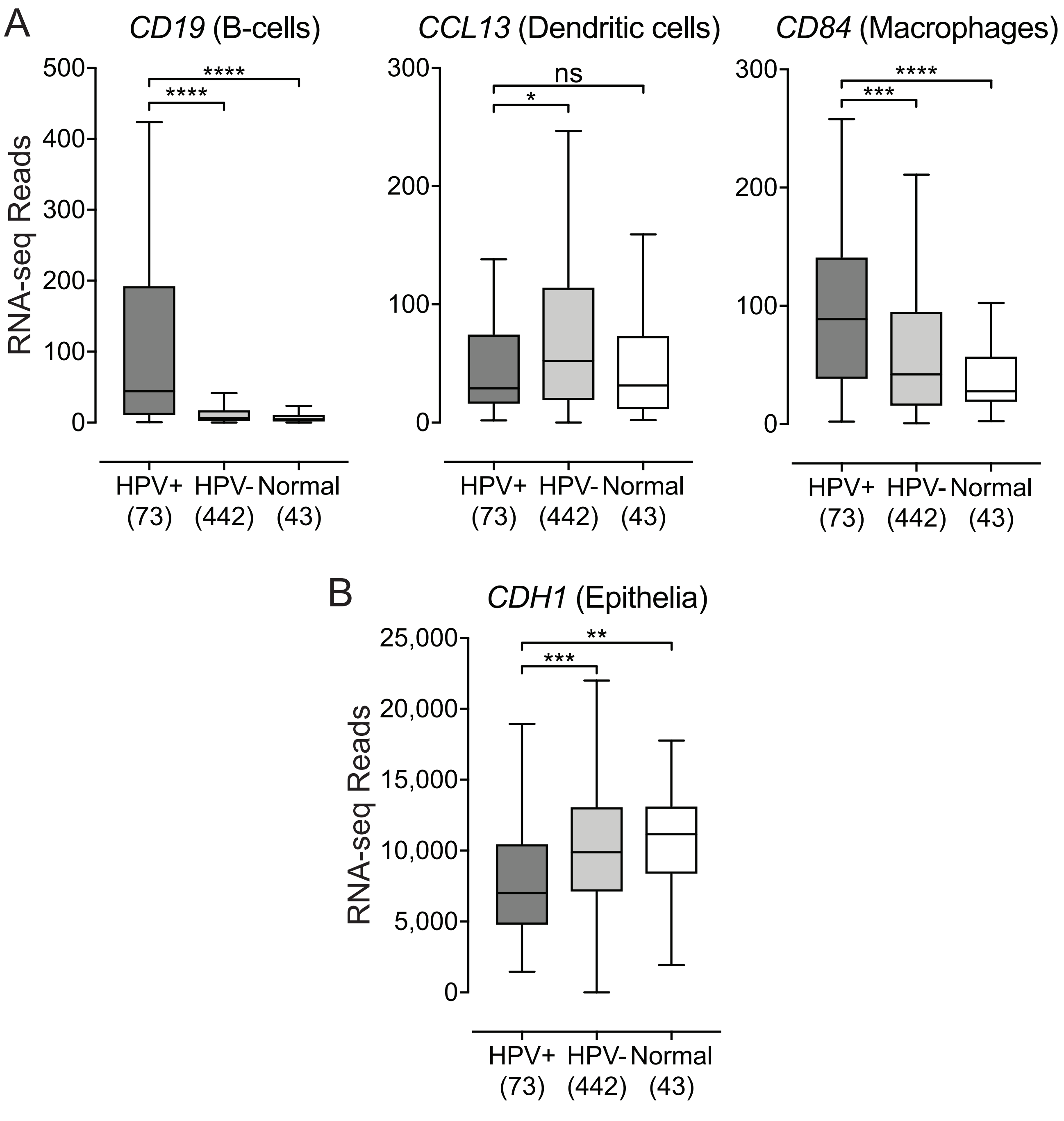

Figure S2: Gene expression comparisons of **(A)** APC and **(B)** Epithelia markers. RSEM normalized RNA-seq data for genes shown above was extracted from the TCGA database for the HNSC cohort for HPV+, HPV–, and normal control tissues. Statistical analysis was performed using a two-tailed non-parametric Mann-Whitney U test. Numbers in brackets refer to the number of samples included in each analysis. * $p \leq 0.05$, ** $p \leq 0.01$, *** $p \leq 0.001$, **** $p \leq 0.0001$, ns - not significant
